# Office-based vs. operating room-performed laryngopharyngeal surgery: a review of cost differences

**DOI:** 10.1007/s00405-019-05617-z

**Published:** 2019-09-05

**Authors:** Anouk S. Schimberg, David J. Wellenstein, Eline M. van den Broek, Jimmie Honings, Frank J. A. van den Hoogen, Henri A. M. Marres, Robert P. Takes, Guido B. van den Broek

**Affiliations:** 1 Department of Otorhinolaryngology and Head and Neck Surgery, Radboud University Medical Center, Postbus 9101, 6500 HB Nijmegen, The Netherlands; 2 Center for Health Services Research, Larner College of Medicine, University of Vermont, Burlington, USA

**Keywords:** Office-based, Topical anesthesia, Operating room, Pharynx surgery, Larynx surgery, Costs, Cost-effectiveness

## Abstract

**Purpose:**

Office-based transnasal flexible endoscopic surgery under topical anesthesia has recently been developed as an alternative for transoral laryngopharyngeal surgery under general anesthesia. The aim of this study was to evaluate differences in health care costs between the two surgical settings.

**Methods:**

PubMed, EMBASE and Cochrane Library were searched for studies reporting on costs of laryngopharyngeal procedures that could either be performed in the office or operating room (i.e., laser surgery, biopsies, vocal fold injection, or hypopharyngeal or esophageal dilation). Quality assessment of the included references was performed.

**Results:**

Of 2953 identified studies, 13 were included. Quality assessment revealed that methodology differed significantly among the included studies. All studies reported lower costs for procedures performed in the office compared to those performed in the operating room. The variation within reported hospital and physician charges was substantial.

**Conclusion:**

Office-based laryngopharyngeal procedures under topical anesthesia result in lower costs compared to similar procedures performed under general anesthesia.

**Electronic supplementary material:**

The online version of this article (10.1007/s00405-019-05617-z) contains supplementary material, which is available to authorized users.

## Introduction

Most patients with lesions in the pharynx or larynx are traditionally treated in the operating room (OR) under general anesthesia. Since flexible endoscopes with distal chip technology have been introduced, the imaging quality improved significantly compared to fiber optic images. As a result, it has become easier to assess lesions in the laryngopharyngeal region. The recent incorporation of a working channel into digital transnasal video-endoscopes facilitates diagnostic and treatment-related procedures. The transnasal approach used during flexible endoscopy results in more surgical control and better patient tolerability than traditional transoral techniques [1]. By eliminating the need for general anesthesia, possible adverse events associated with general anesthesia are averted and the diagnostic and therapeutic phases are accelerated [2].

Over the past 2 decades, several reviews have been conducted on the development of procedures in the office [1, 3-7]. Office-based (OB) procedures such as vocal fold injection (VFI), transnasal flexible laser surgery (TNFLS) [8], flexible endoscopic laryngopharyngeal biopsies (FEB), and transnasal esophagoscopy (TNE) were demonstrated to be feasible, safe and effective [3, 6]. Transnasal esophageal balloon dilation (TNE-BD) has emerged more recently and is a safe and tolerable procedure as well [3]. However, these techniques are not yet widely implemented. A possible explanation might be the financial investment in equipment that is required before the OB procedures can be performed. Furthermore, inadequate reimbursement has been suggested as a principal barrier to the widespread adoption of OB laryngopharyngeal procedures by several authors, since they may not cover all costs [9-11].

Reimbursement is largely dependent on price negotiations between insurer and provider or hospital, as well as the combined clinical and economic value of the treatment. Therefore, it is important to get a better understanding of cost savings from OR-based laryngopharyngeal procedures to OB procedures, to advice decision-makers regarding adequate reimbursement levels. Even though the actual costs associated with the implementation and practice of OB surgery are context-specific, there is growing evidence to support that OB procedures can be performed at lower costs. This study aims to clarify these cost differences by presenting a systematic review of the relevant literature comparing the costs of laryngopharyngeal surgery performed in the office and the OR.

## Methods

A literature search was conducted in three electronic databases (i.e., PubMed, EMBASE and Cochrane Library) in December 2017, and updated in June 2019. A research protocol was developed prior to the start of the review, which was published on the PROSPERO website on May 1st, 2018 [12]. Keywords for the search query included “pharynx”, “larynx”, combined with “ambulatory surgical procedures” or “surgical procedures”, combined with “costs”. A variety of synonyms of these keywords was included in the query (Appendix 1). The aim was to identify all studies that reported on costs of laryngopharyngeal procedures that could either be performed in an OB setting under topical anesthesia, or in an OR setting under general anesthesia (GA). Exclusion criteria were studies not aimed at laryngopharyngeal surgery, not reporting on procedures that could be performed both office-based under topical anesthesia or in the OR under GA, not reporting on a cost-analysis, or not available in English or Dutch. Furthermore, review articles, conference abstracts and animal studies were excluded.

**Fig. 1:**
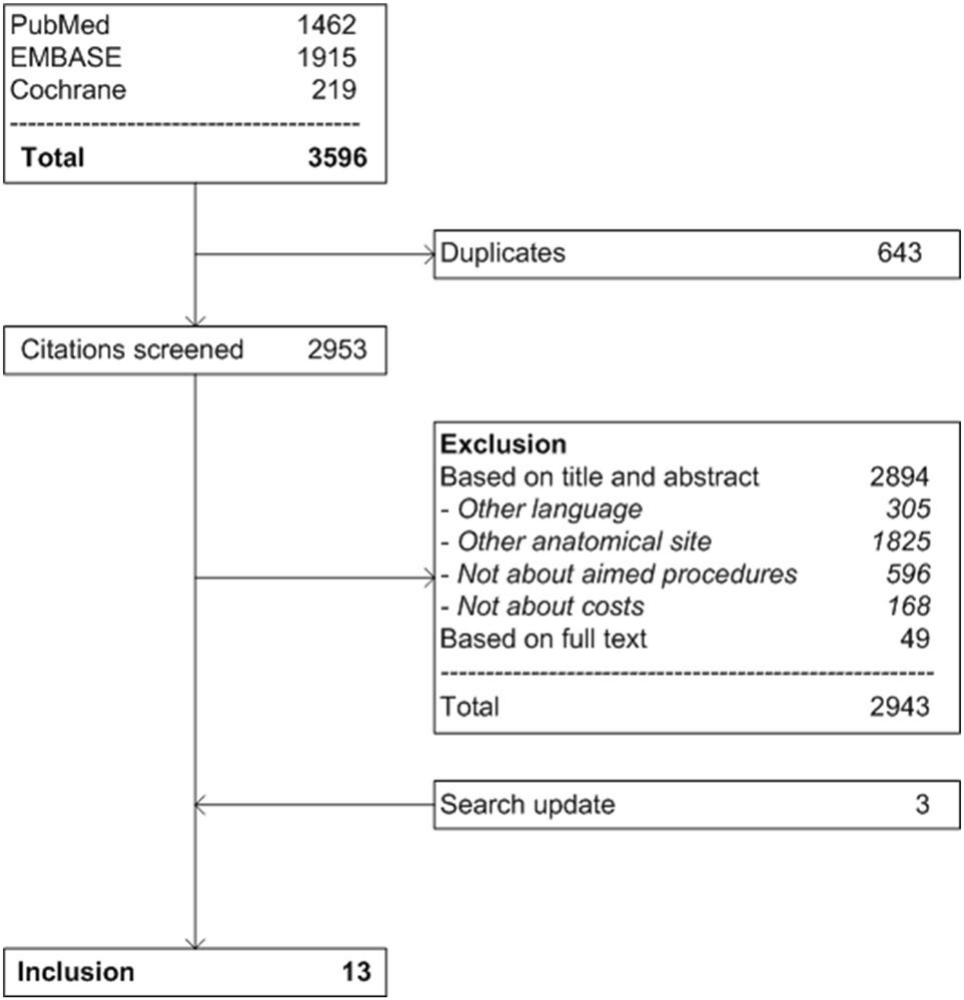
Flow chart of study selection

First, duplicate references were removed from the search results (Fig. 1). Two authors (AS and DW) independently assessed articles on their eligibility. Full texts were retrieved and screened if an article was potentially eligible. In case of disagreement between the two assessors, a third author (GB) was consulted and consensus was reached. Data extraction included the following: author, year of publication, country, journal, number of financially analyzed cases, study design, intervention, pathology of studied subjects, perspective of financial analysis, sources and types of financial data, time frame in which financial data were collected, costs items included in financial analyses, currency and study outcomes.

In any analysis of comparative costs of alternative treatments, study outcomes must be an accurate reflection of the economic comparison. Some studies in the past decades, particularly those performed in the United States, have focused on patient bills (charges) as a proxy for costs, but it is important to make a distinction between the economic cost, accounting cost, and charges to the patient, and actual resource consumption [13]. Charges are the individual list prices a hospital must set, usually for administrative purposes, which do not relate to the payments hospitals actually receive. Payments that hospitals receive are based on negotiations between the hospital or provider and the payer (private insurance or government scheme). Payments are not always a direct reflection of actual costs either, they are based on price agreements and negotiated discounts, reflected in claims data. Costs consist of overhead costs for the hospital, the allowance for differential timing of costs, and the role and estimation of productivity costs [14]. In studies in the US, these are usually divided into facility costs and physician costs. Charges usually do not include physician costs.

Many studies use methods to adjust market prices or hospital charges, most often cost-to-charge ratios. But the way the ratios are being calculated differs widely, including converting hospital charges to cost by use of hospital-level cost-to-charge ratios [15] or department-specific ratios [16]. An important conclusion from that literature is that, while there are significant differences in the magnitude of the estimates obtained by these different methods, the method used to estimate costs did not affect the main results of the economic comparison [14]. Therefore, we chose a similar method for our economic comparison of the selected studies, where we classified the outcomes into: (1) hospital costs, if possible divided into subcategories of materials, equipment (costs for purchase, depreciation, sterilization, and maintenance), facility costs, supporting departments and overhead costs; (2) hospital charges, and (3) physician costs or fees. As mentioned, the proportion of charges that is covered by the insurer depends on agreements and contracts between the hospital and third party payers. Actual hospital costs are thus a better approach of the value of the provided service than charges, and therefore the focus of this review was set on these costs. Physician fees can also be subject to local agreements, therefore this category was separately evaluated.

In case important information was missing in the included articles (e.g., an itemized list of costs or charges), the corresponding author was consulted. In some cases, additional data were sent and used in our cost calculations. Therefore, the amounts displayed in this review can differ from the amounts displayed by the original articles. Additionally, some authors indicated that costs were an equivalent of charges in their health care system. Hence, charges displayed in those studies were documented as costs, as those amounts still reflected the (approximate) actual value of the provided service.

To eliminate currency variability and thereby improve the comparability of study outcomes, costs were converted into Euros (IMF data set, reference country the Netherlands, reference year 2019) using the CCEMG-EPPI-Centre Cost Converter. This converter accounted for currency conversion as well as inflation factors [17]. When a range of years was described in the included article, the median was used for cost conversion. In case the time frame in which the costs were made was not stated, the year of publication was used for the cost conversion. In addition, relative cost differences (%) between office-based and operating room-procedures were calculated per study by dividing the costs of the OB-procedure by the costs of the OR-procedure.

Two authors (AS and DW) independently assessed the methodological quality of the included references. The BMJ checklist for economical evaluations [18] and the CHEC (Consensus Health Economic Criteria) [19] was adapted and used for the assessment. The quality criteria that were addressed can be found in Table 1.

**Table 1:** Quality assessment

	Andrade Filho et al. [23]	Bové et al. [24]	Castillo Farias et al. [25]	Chandran et al. [26]	Fang et al. [10]	Hillel et al. [27]	Hogikyan et al. [28]	Howell et al. [20]	Kuo et al. [9]	Naidu et al. [29]	Rees et al. [11]	Schutte et al. [21]	Wellenstein et al. [22]
1. Is the intervention clearly described?	+	+	+	+	+	−	+	+	+	+	+	+	+
2. Are cost items clearly described?	−	−	−	+	+	+	+	+	+	+	+	+	+
3. Are all relevant cost items included?	?	?	−	+	+ / −	+	−	+	+	+ / −	+	+	+
4. Is an adequate source of financial data used?	+	+	+	+	+	+	?	+	+	?	+	+	+
5. Are productivity changes reported?	−	−	−	−	−	+	−	−	−	−	−	−	−
6. Is the time frame stated?	−	+	+	+	+	+ / −	+	+	+	+	+	+	+
7. Is a sensitivity analysis performed?	−	−	−	−	−	−	−	−	−	−	−	−	−
8. Are appropriate statistical methods used?	NA	+	NA	+	+	+ / −	NA	+	NA	+	NA	NA	+
9. Is discounting applied?	−	−	−	−	−	−	−	−	−	−	−	−	−
10. Conflict of interests that could influence results? (+ = no; − = yes or not stated)	−	−	−	+	+	+	−	+	+	−	−	+	+

+, yes; −, no; +/−, yes, but incomplete or insufficient; ?, not described; NA, not applicable

## Results

### Search results and study selection

The search strategy yielded a total of 2953 unique articles (Fig. 1). After excluding 2894 articles based on title and abstract, 59 articles were considered potentially eligible and full text was retrieved. From this selection, another 49 articles were excluded because inclusion criteria were not met. The search update yielded three additional articles suitable for inclusion [20-22]. In total, 13 articles were included [9-11, 20-29].

### Data extraction

Table 2 shows a summary of the extracted data from the included articles. All included articles concerned retrospective reviews. All articles used financial data based on actual cases, except for the studies of Castillo Farías et al. [25] and Schutte et al. [21], who estimated costs based on costs of materials, facilities and personnel required to perform these procedures in general. Hospital costs were discussed in seven studies [9, 10, 21, 22, 25-27]; hospital charges in eight studies [9, 11, 20, 23, 24, 27-29]. All included studies reported on costs from a hospital’s perspective. The time frame in which financial data were collected ranged from 1995 to 2018. In the studies of Hillel et al. [27] and Chandran et al. [26], the analyzed costs were about awake surgery performed in the (in-hospital) endoscopy suite or instead of the office. The included parameters for costs or charges differed among studies. For example, some studies explicitly described that purchase costs, write-off and maintenance of technical equipment, such as for video-endoscopes with working channel, were incorporated in the cost-analysis [9, 20-22, 26]. Two studies did not specify the items included in the costs analysis [23, 24]. One study did not include physician fees in the cost-analysis, but focused rather on facility costs [20]. Table 3 demonstrates the number of studies that addressed the costs of the different procedures.

**Table 2:** Extracted data of included studies (*n* = 13)

Author, year, country	Pathology of study population	Intervention	Perspective	Source and included items of OB costs/charges	Source and included items of OR-costs/charges	Results converted to Euros
Andrade Filho [23], 2006, USA	Glottal insufficiency	OB-VFI *n* = 52 OR-VFI *n* = 10	Hospital charges	Office billing records: not further specified	Hospital billing records: not further specified	OB charges: €1.212 OR charges: €12.049
Bové [24], 2007, USA	Vocal fold atrophy, paresis or paralysis	OB-VFI *n* = 50 OR-VFI *n* = 108	Hospital charges	Hospital billing records: not further specified	Hospital billing records: not further specified	OB charges: €1.472 OR charges: €12.430 (*p* < 0.05)
Castillo Farias [25], 2014, Spain	Suspected pharyngo-laryngeal malignancy	FEB and direct laryngoscopy biopsies under GA	Hospital costs	Financial department of hospital (based on general fees): medical supplies, biopsy/cytology	Financial department of hospital (based on general fees): pre-operative examinations, pre-anesthesiology visit, surgery and related costs, pathology	OB costs: €57 OR costs: €1.101
Chandran [26], 2017, Australia^a^	Vocal fold paralysis	OB-VFI *n* = 14 (in OR) OR-VFI *n* = 6 ×	Hospital costs	Financial department of the hospital: ward costs (medical, nursing, and supplies), operating theatre costs (theatre nursing, surgeon, and anesthetic), allied health, depreciation, overhead expenditures, hotel, non-clinical costs, in-patient admissions, outpatient visits, emergency department visits, pathology, radiology, medication, nursing, and medical staff, associated overheads		OB costs: €980 OR costs: €1.622 *p* < 0.001
Fang [10], 2015, Taiwan	Pharyngo-laryngeal lesion	FEB (*n* = 20) and direct laryngoscopy biopsies under GA (*n* = 20)	Hospital costs	National Taiwan health insurance program: physician fee, flexible laryngoscopy, disposable supplies, biopsy procedure fee	NA	OB costs: Hospital €13 Surgeon €97
Hillel [27], 2016, USA^b^	Not stated	OB-VFI *n* = 16 (in endoscopy suite) OR-VFI *n* = 16	Hospital costs and charges	Hospital billings records: pre-operative unit laboratory testing, intravenous fluids, drugs administered, laser fiber or tissue filler, pathology charges, room charges, recovery charges, surgeon fees and anesthesiologist fees (OR-cases only)		OB costs: Hospital €1.456 OB charges: Hospital €2.381 Surgeon €891 OR costs: Hospital €1.870 OR charges: Hospital €6.205 Surgeon €660 Anesthesiologist €661 *p* = 0.0032 (for costs difference OB vs. OR)
	Respiratory papillomatosis	TNFLS *n* = 16 × (in endoscopy suite) TOLMS *n* = 16	Hospital costs and charges	Hospital billings records: pre-operative unit laboratory testing, intravenous fluids, drugs administered, laser fiber or tissue filler, pathology charges, room charges, recovery charges, surgeon fees and anesthesiologist fees (OR-cases only)		OB costs: Hospital €1.228 OB charges: Hospital €2.464 Surgeon’s fee €1.006 OR costs: Hospital €2.292 OR charges: Hospital €7.589 Surgeon €759 Anesthesiologist €1.135
Hogikyan [28], 2000, USA	Suspected pharyngo-laryngeal malignancy	OB-transoral biopsies with rigid laryngoscope (*n* = 2), direct laryngoscopy biopsies under GA (*n* = 2)	Hospital charges	Billing records: laryngoscopy, surgeon fee	Billing records: laryngoscopy, esophagoscopy, surgeon fee, anesthesia/OR	OB charges: Hospital €579 OR charges: Hospital €3.583
Howell [20], 2018, USA	Esophageal stenosis in patients with HNCA history and dysphagia	OB-TNE balloon dilation (*n* = 9); OR-Esophageal dilation (*n* = 9)	Hospital charges	Billing records: clinic visit, outpatient services, speech therapy (physicians fees not included)	Billing records: anesthesia, laboratory, pathology, supplies and devices, OR services, pharmacy, pulmonary function, recovery room, respiratory services, room and board, speech therapy (physician fees not included)	OB charges: Hospital €2.738 OR charges: Hospital €15.090 *p* < 0.001
Kuo [9], 2011, USA	Benign laryngeal pathology	TNFLS *n* = 6 TOLMS *n* = 6	Hospital costs and charges	Hospital billings records: disposable supplies and other materials, anesthesia and medications, equipment, staff, procedural room-related fees, surgeon fee, recovery room and anesthesiologist fee (OR-cases only)		OB costs: Hospital €1.239 OB charges: Hospital €187 Surgeon €946 OR costs: Hospital €1.847 OR charges: Hospital €10.311 Surgeon €1.249 Anesthesiologist €1.287
Naidu [29], 2011, USA	Suspected pharyngo-laryngeal malignancy	FEB (*n* = 12) and direct laryngoscopy biopsies under GA (*n* = 5)	Hospital charges	Financial department of hospital: facility, procedure charges, and professional otolaryngology charges	Financial department of hospital: anesthesia charges, OR overheads, surgeon fee and postoperative care	OB charges: Facility €173 Procedure €1.248 Surgeon €455 OR charges: Facility €5.752 Surgeon €435 Anesthesia (incl anesthesiologist’s fee) €2.056 *p* = 0.0001
Rees [11], 2007, USA	Respiratory papilloma-tosis	TNFLS *n* = 7 TOLMS *n* = 6	Hospital charges	Hospital billings records: drugs, supplies, laser charges, office visit, surgeon fee	Hospital billing records: pre-operative assessments, i.v. fluids, drugs, supplies, laser charges, OR, anesthesia, post-anesthetic care unit, anesthesiologist and surgeon fee	OB charges: Hospital €249 Surgeon €1.796 OR charges: Hospital €3.677 Surgeon €3.177 Anesthesiologist €814
Schutte [21], 2018, the Netherlands	Suspected pharyngo-laryngeal malignancy	FEB and direct laryngoscopy biopsies under GA	Hospital costs	Financial department of hospital (based on general fees): purchase, depreciation, sterilization of equipment, costs of disposables, salary of involved medical employees	Financial department of hospital (based on general fees): purchase, depreciation, sterilization of equipment, costs of disposables, salary of involved medical employees, institutional rates for day-care	OB costs: Hospital €54 Physician €25 OR costs: Hospital €340 Surgeon €41 Anesthesiologist (incl. OR facility fee) €440
Wellenstein [22], 2019, the Netherlands	Hypopharyngeal carcinoma	TNE-assisted biopsies (*n* = 15) and direct laryngoscopy biopsies under GA (*n* = 15)	Hospital costs	Financial department of hospital (based on general fees): purchase, depreciation, sterilization of equipment, costs of disposables, outpatient visits, salary of involved medical employees	Financial department of hospital (based on general fees): purchase, depreciation, sterilization of equipment, costs of disposables, outpatient visits, salary of involved medical employees (excl. ENT surgeon), institutional rates for day-care	OB costs: Hospital including physician: €583 OR costs: Hospital €975 Anesthesiologist (incl. OR facility fee) €440 *p* = 0.000

*OB *office-based, *OR* operating room, *GA* general anesthesia, *VFI* vocal fold injection, *FEB* flexible endoscopic biopsy, *TNFLS* transnasal flexible laser surgery, *TOLMS* transoral laser microsurgery, *TNE* transnasal esophagoscope

^a^Awake laryngeal surgery performed in the operating theatre; ^b^awake laryngeal procedures performed in the endoscopy suite

**Table 3:** The number of included studies addressing costs of several types of office-based surgery

Procedure	Number of studies addressing costs of this procedure
Vocal fold injection (VFI)	4 [4, 23, 26, 27]
Transnasal flexible laser surgery (TNFLS)	3 [1, 7, 9]
Flexible endoscopic (laryngopharyngeal) biopsies (FEB)	5 [1, 5, 8–10]
Transnasal esophageal balloon dilation (TNE-BD)	1 [20]
Transnasal esophagoscope-assisted biopsies (TNE-B)	1 [2]

### Quality assessment

The quality assessment (Table 1) illustrates that none of the included articles implemented a formal cost-analysis, as can be understood from the absence of sensitivity analyses, reports on productivity changes, and discounting. In many studies the included costs were lacking essential items, such as costs for equipment and overhead costs. As a result most estimates will include measurement error, varying in both magnitude and direction.

### Hospital costs

**Fig. 2:**
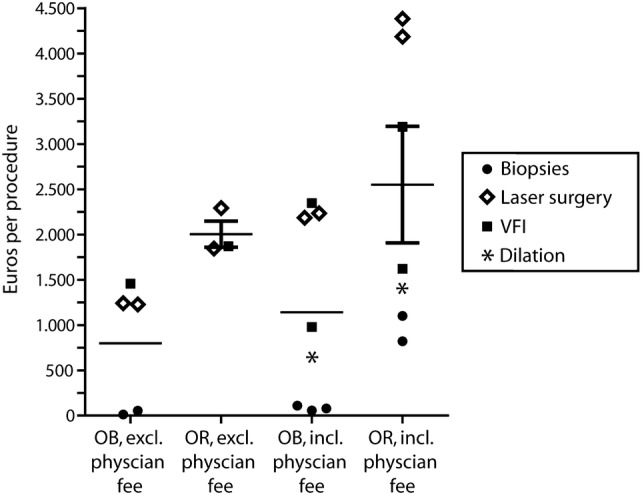
Hospital costs (with and without physician fees) for office-based and OR-performed procedures in Euros per procedure

All included studies reported that hospital costs for OB procedures were reduced compared to similar procedures performed in the OR. The relative reduction in hospital costs varied from 22% [27] to 46% [27] (Fig. 2). Because hospital costs were not always presented separately from physician fees, total hospital costs including physician fees are also depicted. The relative reduction in total hospital costs including physician fees ranged from 27% [27] to 95% [25] per procedure. Specifically, for VFI procedures, total costs ranged from €980 [26] to €2.347 [27] when performed in an OB setting, and from €1.622 [26] to €3.191 [27] when performed in the OR. Costs of OB TNFLS ranged from €2.185 [9] to €2.234 [27], and for OR-performed TNFLS costs ranged from €4.186 [27] to €4.383 [9]. Costs of FEB ranged from €57 [25] to €110 [10], whereas costs for biopsies obtained under GA varied between €822 [21] and €1.101 [25]. Costs for the diagnostic trajectory in hypopharyngeal carcinoma were €583 when performing TNE-assisted biopsies compared to €1415 when performing biopsies under general anesthesia [22].

### Hospital charges

**Fig. 3:**
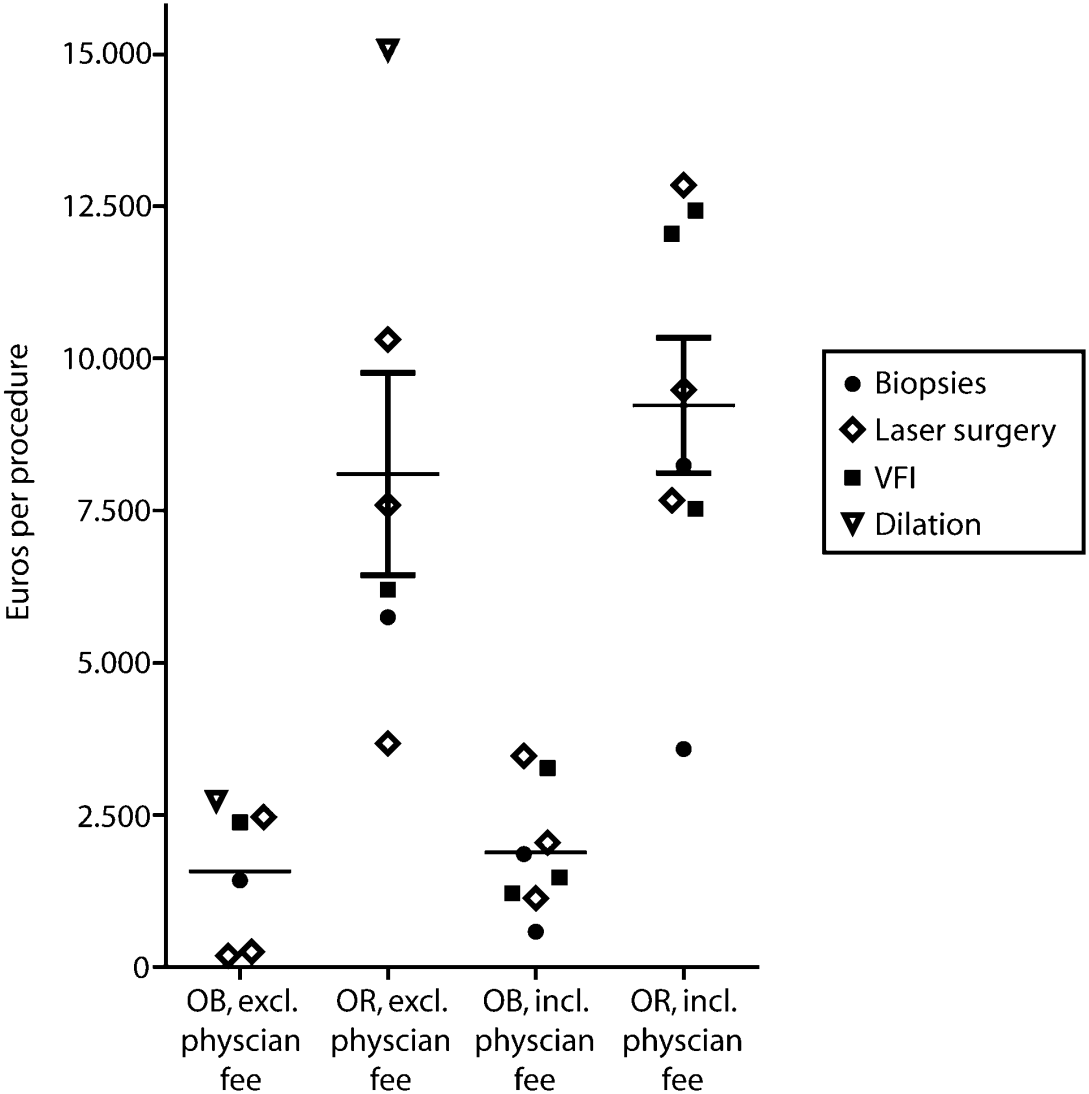
Hospital charges (with and without physician fees) for office-based and OR-performed procedures in Euros per procedure

All included studies revealed that hospital charges for OB procedures were reduced compared to similar procedures performed in the OR. The relative reduction in hospital charges varied from 62% [27] to 98% [9] (Fig. 3). The relative reduction in hospital charges including physician fees ranged from 73% [11] to 89% [23]. By comparing Figs. 2 and 3, the large differences in costs and charges can be deducted. Additionally, a substantial variation existed within hospital charges, especially in OR-performed procedures (€3.677 [11]–€15.080 [20]).

### Physician fees

**Fig. 4:**
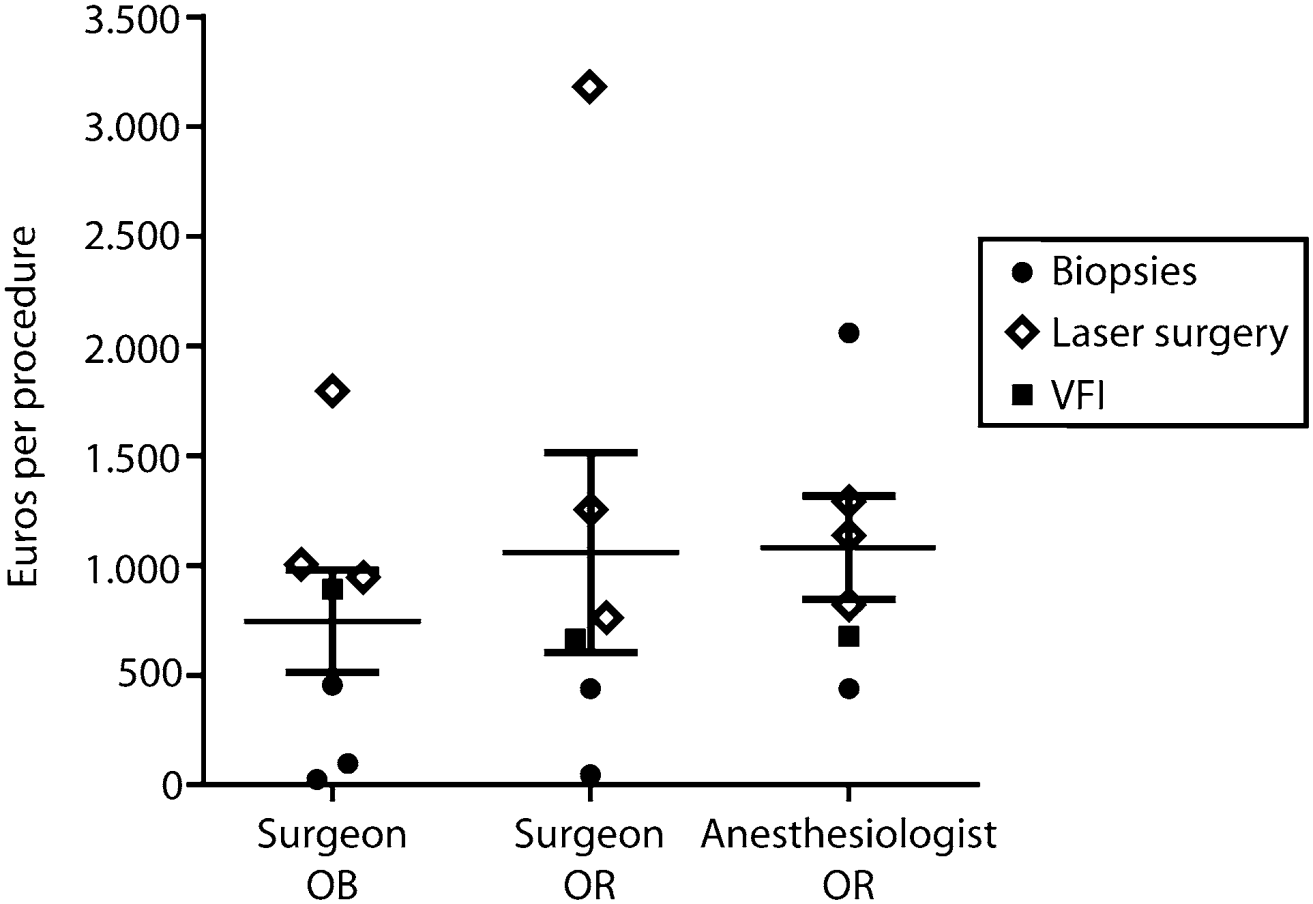
Physician fees for office-based and OR-performed procedures in Euros per procedure

Except for charges reported in one study [11], otorhinolaryngologist fees for OB and OR-performed procedures were comparable (Fig. 4). Still, a wide variation in physician fees existed, i.e., €25 [21]–€1.796 [11] per OB procedure and €41 [21]–€3.179 [11] per OR procedure. As anesthesiologists are not involved in OB procedures, total physician charges in OB procedures are lower than OR-performed procedures.

## Discussion

This study identified and evaluated differences in health care costs between the OB and OR-performed laryngopharyngeal surgery. By reviewing the relevant literature between 1995 and 2019, it was demonstrated that significant cost savings can be realized by shifting laryngopharyngeal procedures from the OR to the outpatient clinic.

In this review, clear distinctions were made between costs and charges, because costs do not correspond with charges. It is important to consider that health care providers in certain countries tend to inflate charges for their services, as third party payers cover only small proportions of the billed amounts [30]. This phenomenon also follows from our results, in which hospital charges are much higher than the actual costs incurred by the hospital in providing the service. On the other hand, cost analyses are often incomplete as important cost items are not always included, while charges obtained from billing records assumedly consist of full charges. There is also variation in cost-to-charge ratios within one hospital or health system which is not always transparent.

This review revealed that procedures under topical anesthesia resulted in hospital cost reductions of up to 95% per procedure compared to similar procedures under GA. However, the total costs per procedure varied widely between studies. Several reasons for variations in cost outcomes can be outlined. First, the investigated procedures require different equipment or materials. For example, flexible endoscopic laser surgery requires an expensive laser fiber, while flexible biopsy forceps for FEB are significantly less costly. In addition, the cost items that were involved in the analyses differed between the included studies. Differences existed in whether pre-operative general examinations, purchase and use of expensive equipment, or overhead costs were incorporated. Costs for learning these new surgical techniques (e.g., costs for courses and prolonged surgery duration) were not considered in any of the studies. Third, two of the included studies described that awake surgery was performed in the endoscopy suite [27] or in the OR [26], instead of the (non-facility based) office, to bypass reimbursement problems for in-office procedures. Both reports described the least cost reductions among the included articles. Endoscopy suites or ORs, even though no anesthesiologist is involved during the procedure, are associated with higher facility charges than the outpatient office. For practitioners who want to start performing OB laryngopharyngeal surgery, but find safety issues prohibitive, using the endoscopy suite or can be helpful as these settings usually provide adequate materials and staff.

Possible predictors of costs per procedure were analyzed by one study. Chandran et al. described that time spent in the operating theatre was a significant predictor for total costs, but when time was excluded from the multiple linear regression model, type of anesthesia emerged as an independent predictor of total costs [26]. These findings reveal that topical anesthesia is directly linked with reduced costs.

The opacity of financial data in current health care systems around the world is challenging in this type of research and leads to limitations. The costs of delivering health care are obscured in layers of jargon and complex accounting [30]. Hence, it is difficult to gain full insight in the financial aspects of provided health care services. Additionally, differences between countries in organization and financing of health care, and diverging methodology among the included studies lead to difficulties in comparability of the study outcomes. Even within countries, differences between health care organizations can lead to external validity and generalizability issues. Taking this into account, we improved comparability as much as possible by converting financial data of the included studies into Euros and correcting for inflation over years. Still, due to the diverging methodology, statistical (meta-)analysis was not appropriate to perform in this review.

There is an opportunity for cost-effectiveness studies comparing laryngopharyngeal surgery in the office to transoral laryngopharyngeal surgery under general anesthesia, as costs from societal or patient perspective are not yet available. Taking into account the reduced sedative effect when topical anesthesia is used instead of general anesthesia, allowing patients to return to work even the same day as the procedure, costs from patients’ perspectives can be significantly diminished. Based on our findings, indications exist that performing laryngopharyngeal surgery in the office under topical anesthesia instead of the OR under GA results in significant costs savings for the hospital and third party payer. Other fields of medicine have also shown cost reductions in shifting surgical interventions to the office [31-34]. To gain full understanding of costs involved in these procedures, broad multi-center cost-effectiveness studies are necessary.

In conclusion, the current review demonstrates that shifting laryngopharyngeal procedures from the OR to the office results in apparent economic benefits. From a physician’s point of view, deciding whether to start performing office-based procedures will not only depend on costs, but also on training of the surgeon and other involved staff and availability of materials, instruments and facilities. The results of this review contribute to a better understanding of cost savings from shifting OR-based laryngopharyngeal procedures to the office and can reinforce physicians who are planning to make this shift. Furthermore, the results can be used to guide decision-makers regarding adequate reimbursement levels.

## Electronic supplementary material

Below is the link to the electronic supplementary material.

Supplementary file1 (DOCX 14 kb)
