# Supplementary material for: Office-based vs. operating room-performed laryngopharyngeal surgery: a review of cost differences
Source: Eur Arch Otorhinolaryngol. 2019 Sep 5;276(11):2963–73. doi: 10.1007/s00405-019-05617-z (PMC6811667; doi:10.1007/s00405-019-05617-z)
Supplement: Supplementary file 1 — Supplementary file1 (DOCX 14 kb) [file 405_2019_5617_MOESM1_ESM.docx]

**Appendix 1. Search queries**

1. **PubMed**

("Esophageal Neoplasms"[Mesh] OR "Laryngeal Diseases"[Mesh] OR "Pharyngeal Diseases"[Mesh:NoExp] OR "Pharyngeal Neoplasms"[Mesh] OR ((Cancer*[tiab] OR Neoplas*[tiab] OR Tumor*[tiab] OR Tumour*[tiab] OR Malignan*[tiab]) AND (Esophagus[tiab] OR Oesophagus[tiab] OR Esophageal[tiab] OR Oesophageal[tiab] OR Tonsil[tiab] OR Tonsillar[tiab] OR Oropharynx[tiab] OR Oropharyngeal[tiab] OR Hypopharyngeal[tiab] OR Hypopharynx[tiab] OR Pharyngeal[tiab] OR Pharynx [tiab]OR Laryngeal[tiab] OR Larynx[tiab] OR glottic[tiab] OR glottis[tiab] OR subglottic[tiab] OR subglottis[tiab] OR supraglottic[tiab] OR supraglottis[tiab])) OR ((laryngeal[tw] OR larynx[tw] OR pharynx[tw] OR pharyngeal[tw]) AND (edema[tiab] OR granuloma[tiab] OR keratosis[tiab] OR nerve injur* [tiab] OR papillomatosis[tiab] OR paralysis[tiab] OR paresis[tiab] OR stenosis[tiab])) OR laryngocele[tiab] OR laryngomalacia[tiab] OR laryngostenosis[tiab] OR glottic[tiab]OR glottis[tiab] OR subglottic[tiab] Or subglottis[tiab] OR supraglottic[tiab] OR supraglottis[tiab] OR vocal cord [tiab] OR vocal fold[tiab] OR aphonia[tiab] OR dysfonia[tiab] OR hoarseness[tiab]) AND ((“Ambulatory Surgical Procedures"[Mesh] OR Day Surger*[tiab] OR (("Outpatients"[Mesh] OR "Outpatient Clinics, Hospital"[Mesh] OR "Ambulatory Care"[Mesh] OR Ambulatory [tiab] OR Outpatient*[tiab] OR Out-patient*[tiab] OR office-based[tiab] OR in-office[tiab] OR intra-office[tiab] OR Office[tiab]) AND ("Anesthesia, Local"[Mesh] OR "Anesthetics, Local"[Mesh] OR Procedure*[tiab] OR Surger*[tiab] OR Surgical*[tiab] OR local anesthe*[tiab] OR local anaesthe*[tiab] OR topical anesthe*[tiab] OR topical anaesthe*[tiab] OR Infiltration Anesthe*[tiab] OR infiltration anaesthe*[tiab] OR unsedated[tiab] OR endoscop*[tiab] OR transnasal*[tiab] OR laryngoscop*[tiab] OR pharyngolaryngoscop*[tiab]))) OR ("Surgical Procedures, Operative"[Mesh] OR "Anesthesia, General"[Mesh] OR "Operating Rooms"[Mesh] OR surgical procedure*[tiab] OR operative[tiab] OR General Anesthe*[tiab] OR general anaesthe*[tiab] OR Operating Room*[tiab] OR operating theatre*[tiab] OR operating theater*[tiab])) AND (cost[tw] OR costs[tw] OR costs and cost analysis[mh] OR ec[sh] OR reimbursement*[tw] OR financial[tw] OR saving*[tw] OR expenditure*[tw] OR econom*[tw])

1. **EMBASE**

esophagus tumor/ or esophagus cancer/ or larynx disorder/ or respiratory tract disease/ or throat disease/ or hoarseness/ or laryngocele/ or laryngomalacia/ or larynx edema/ or larynx injury/ or larynx stenosis/ or larynx tumor/ or recurrent laryngeal nerve palsy/ or spasmodic dysphonia/ or subglottic stenosis/ or vocal cord disorder/ or vocal cord paralysis/ or pharynx disease/ or throat disease/ or pharynx tumor/ or ((Cancer* or Neoplas* or Tumor* or Tumour* or Malignan*) and (Esophagus or Oesophagus or Esophageal or Oesophageal or Tonsil or Tonsillar or Oropharynx or Oropharyngeal or Hypopharyngeal or Hypopharynx or Pharyngeal or Pharynx or Laryngeal or Larynx or glottic or glottis or subglottic or subglottis or supraglottic or supraglottis)).ab,kw,ti. OR ((laryngeal or larynx or pharynx or pharyngeal) and (edema or granuloma or keratosis or nerve injur* or papillomatosis or paralysis or paresis or stenosis)).ab,kw,ti. OR (laryngocele or laryngomalacia or laryngostenosis or glottis or glottis or subglottic or subglottis or supraglottic or supraglottis or vocal cord or vocal fold or aphonia or dysfonia or hoarseness).ab,kw,ti.

AND

ambulatory surgery/ or (day surger*).ab,kw,ti. OR ((outpatient/ or outpatient care/ or ambulatory care/ or (Ambulatory or Outpatient* or Out-patient* or office)).ab,kw,ti. AND local anesthesia/ or topical anesthesia/ or local anesthetic agent/ or (procedure* or surger* or Surgical* or local anesthe* or local anaesthe* or topical anesthe* or topical anaesthe* or infiltration anesthe* or infiltration anaesthe* or unsedated or endoscop* or transnasal* or laryngoscop* or pharyngolaryngoscop*).ab,kw,ti. OR surgery/ or medical procedures/ or general anesthesia/ or operating room/ or (surgical procedure* or operative or general anesthe* or general anaesthe* or operating room* or operating theatre* or operating theater*).ab,kw,ti.

AND

cost benefit analysis/ or cost effectiveness analysis/ or ec.fs. or (cost or costs or reimbursement* or financial or saving* or expenditure* or econom*).mp.

1. **Cochrane Library**

(Cancer* or Neoplas* or Tumor* or Tumour* or Malignan*:ti,ab,kw) AND Esophagus or Oesophagus or Esophageal or Oesophageal or Tonsil or Tonsillar or Oropharynx or Oropharyngeal or Hypopharyngeal or Hypopharynx or Pharyngeal or Pharynx or Laryngeal or Larynx or glottic or glottis or subglottic or subglottis or supraglottic or supraglottis:ti,ab,kw)

OR MeSH descriptor: [Esophageal Neoplasms] 3 tree(s) exploded OR MeSH descriptor: [Laryngeal Diseases] explode all trees OR MeSH descriptor: [Pharyngeal Diseases] this term only OR MeSH descriptor: [Pharyngeal Neoplasms] explode all trees OR ((laryngeal or larynx or pharynx or pharyngeal) and (edema or granuloma or keratosis or nerve injur* or papillomatosis or paralysis or paresis or stenosis)):ab,kw,ti OR (laryngocele or laryngomalacia or laryngostenosis or glottic or glottis or subglottic or subglottis or supraglottic or supraglottis or vocal cord or vocal fold or aphonia or dysfonia or hoarseness):ab,kw,ti

AND

MeSH descriptor: [Ambulatory Surgical Procedures] explode all trees OR Day Surger*:ti,ab,kw OR MeSH descriptor: [Outpatients] explode all trees OR MeSH descriptor: [Outpatient Clinics, Hospital] explode all trees OR MeSH descriptor: [Ambulatory Care] this term only OR (Ambulatory or Outpatient* or Out-patient* or office-based or in-office or intra-office or Office):ab,kw,ti OR MeSH descriptor: [Anesthesia, Local] explode all trees OR MeSH descriptor: [Anesthetics, Local] explode all trees OR (Procedure* or Surger* or Surgical* or local anesthe* or local anaesthe* or topical anesthe* or topical anaesthe* or Infiltration Anesthe* or infiltration anaesthe* or unsedated or endoscop* or transnasal* or laryngoscop* or pharyngolaryngoscop*):ab,kw,ti OR MeSH descriptor: [Surgical Procedures, Operative] explode all trees OR MeSH descriptor: [Anesthesia, General] explode all trees OR MeSH descriptor: [Operating Rooms] explode all trees OR (surgical procedure* or operative or General Anesthe* or general anaesthe* or Operating Room* or operating theatre* or operating theater*):ab,kw,ti

AND

MeSH descriptor: [Costs and Cost Analysis] explode all trees OR (cost or costs or reimbursement* or financial or saving* or expenditure* or econom*):ti,ab,kw
